# The Efficacy of the Mineralcorticoid Receptor Antagonist Canrenone in COVID-19 Patients

**DOI:** 10.3390/jcm9092943

**Published:** 2020-09-11

**Authors:** Marco Vicenzi, Massimiliano Ruscica, Simona Iodice, Irene Rota, Angelo Ratti, Roberta Di Cosola, Alberto Corsini, Valentina Bollati, Stefano Aliberti, Francesco Blasi

**Affiliations:** 1 Fondazione IRCCS Ca’ Granda Ospedale Maggiore Policlinico, Cardiovascular Disease Unit, Internal Medicine Department, 20122 Milan, Italy; irene.rota85@gmail.com; 2 Dyspnea Lab, Department of Clinical Sciences and Community Health, University of Milan, 20122 Milan, Italy; angelo.ratti.91@gmail.com (A.R.); dicosola.roberta@gmail.com (R.D.C.); 3 Department of Pharmacological and Biomolecular Science, University of Milan, 20133 Milan, Italy; alberto.corsini@unimi.it; 4 EPIGET Lab, Department of Clinical Sciences and Community Health, University of Milan, 20122 Milan, Italy; simona.iodice@unimi.it (S.I.); valentina.bollati@unimi.it (V.B.); 5 IRCCS Multimedica, Sesto San Giovanni, 20099 Milan, Italy; 6 Department of Pathophysiology and Transplantation, University of Milan, 20122 Milan, Italy; stefano.aliberti@unimi.it (S.A.); francesco.blasi@unimi.it (F.B.); 7 Fondazione IRCCS Ca’ Granda Ospedale Maggiore Policlinico, Internal Medicine Department, Respiratory Unit and Cystic Fibrosis Adult Center, 20122 Milan, Italy

**Keywords:** COVID-19, angiotensin-converting enzyme-2, renin–angiotensin–aldosterone system, mineralcorticoid receptor antagonist, aldosterone, canrenone

## Abstract

Background: In COVID-19 patients, aldosterone via angiotensin-converting enzyme-2 deregulation may be responsible for systemic and pulmonary vasoconstriction, inflammation, and oxidative organ damage. Aim: To verify retrospectively the impact of the mineralcorticoid receptor antagonist canrenone i.v. on the need of invasive ventilatory support and/or all-cause in-hospital mortality. Methods: Sixty-nine consecutive COVID-19 patients, hospitalized for moderate to severe respiratory failure at Fondazione Istituti di Ricovero e Cura a Carattere Scientifico (IRCCS) Ca’ Granda Ospedale Maggiore Policlinico of Milan, received two different therapeutic approaches in usual care according to the personal skills and pharmacological management experience of the referral medical team. Group A (*n* = 39) were given vasodilator agents or renin–angiotensin–aldosterone system (RAAS) inhibitors and group B (*n* = 30) were given canrenone i.v. Results: Among the 69 consecutive COVID-19 patients, those not receiving canrenone i.v. (group A) had an event-free rate of 51% and a survival rate of 64%. Group B (given a mean dose of 200 mg/q.d. of canrenone for at least two days of continuous administration) showed an event-free rate of 80% with a survival rate of 87%. Kaplan–Meier analysis for composite outcomes and mortality showed log rank statistics of 0.0004 and 0.0052, respectively. Conclusions: The novelty of our observation relies on the independent positive impact of canrenone on the all-cause mortality and clinical improvement of COVID-19 patients ranging from moderate to severe diseases.

## 1. Introduction

Since early December 2019, when the first pneumonia cases of unknown origin were identified in Wuhan (China), almost 11.5 million people across the globe have contracted severe acute respiratory syndrome coronavirus 2 (SARS-CoV-2). As of 31 August, SARS-CoV-2 has led to 852,758 deaths worldwide [1]. Despite the spread of this global pandemic and the high volume of clinical trials launched, no consensus in a standardized treatment has been reached yet [2,3].

The foremost hypotheses describing the pathway through which SARS-CoV-2 enters the cells is related to the involvement of angiotensin-converting enzyme-2 (ACE2). Although this enzyme has been described as the receptor for coronaviruses [4], upregulation of ACE2 due to ACE inhibitors (ACEIs) or angiotensin receptor blockers (ARBs) has not been consistently demonstrated in human and animal studies (reported in [5]). ACE2 serves a counterbalancing role in the renin–angiotensin–aldosterone system (RAAS), i.e., it cleaves away (i) phenylalanine from angiotensin (Ang) II, converting it to Ang-(1-7), and (ii) leucine from Ang I, converting it to Ang-(1-9). SARS-CoV-2 infection appears to downregulate ACE2, possibly making the enzyme unable to exert protective effects in organs, e.g., a cardioprotective effect [6]. Indeed, besides coronavirus infections, potentially deleterious effects of RAAS have been reported in heart and lung and medical conditions such as hypertension, heart failure, and obesity [7]. Recent evidence from our group and others shows that progressive activation of the Ang II/angiotensin type 1 receptor (AT1R) axis, along with the final effector—aldosterone—may be responsible for systemic and pulmonary vasoconstriction, inflammation, and oxidative organ damage [8,9,10]. However, in COVID-19 infections, the role of aldosterone has not been disentangled alongside consideration of the beneficial effects observed from anti-aldosterone and RAAS blockers under experimental conditions of pulmonary diseases [11].

In line with the findings that aldosterone levels are associated with severe clinical outcomes in COVID-19 patients [12], we postulated the hypothesis that mineralcorticoid receptor antagonist (MRA) could have a beneficial effect on RAAS activation during SARS-CoV-2 infection. Thus, the present study aimed to verify, in COVID-19 patients, the impact of canrenone i.v. on the need for invasive ventilatory support and/or all-cause in-hospital mortality.

## 2. Experimental Section

### 2.1. Population Description, Treatments, and Biochemical Evaluation

In this retrospective study (CARDIOVID-19, ID 1676), a series of 69 consecutive patients, enrolled in COVID-19 registry network was considered. All patients were hospitalized for severe respiratory failure due to SARS-CoV-2 infection and were cared for at the Cardiorespiratory Sub-Intensive Unit of Fondazione IRCCS Ca’ Granda Policlinico Hospital of Milan. All patients received usual care treatment according to local protocols (hydroxychloroquine, lopinavir/ritonavir, anakinra, and methylprednisone). However, according to the referral physicians’ experience and their skills in pharmacological management, once arterial hypertension (systolic pressure ≥140 mmHg or diastolic pressure ≥90 mmHg) and/or hypokalemia ([K+] <4.0 mmol/L) were manifested [9], patients could have received anti-hypertensive agents and potassium supplementation per os or i.v. (group A) or canrenone i.v. associated with anti-hypertensive agents, if necessary (group B). Thus, the choice of treatment was not based on a randomized design and the experimental groups were composed a posteriori.

Before treatment and during hospitalization, blood pressure, the partial pressure of oxygen to inspiratory fraction of oxygen ratio (PaO_2_/FiO_2_), and alveolar–arterial oxygen gradient (ΔA-aO_2_) were recorded. C-reactive protein (CRP), interleukin-6 (IL-6), D-dimer, and ferritin were determined by blood sample collection during routine laboratory analysis. Composite outcomes as invasive ventilatory support and/or all-cause in-hospital mortality were considered during in-hospital follow-up.

### 2.2. Statistical Method

For normally-distributed demographics and clinical characteristics, data were expressed as the mean and standard deviation; otherwise, they were expressed as the median and interquartile range. Frequencies and percentages were calculated for categorical variables. The differences between the two groups defined as receiving or not receiving canrenone i.v. were compared using Pearson’s chi-square test or Fisher’s exact test for categorical data, or Welsch’s *t*-test or Mann–Whitney’s *U*-test for continuous variables, as appropriate. The effect size of MRA treatment was evaluated with Cohen’s d for unequal group sizes. Survival time was calculated starting at the date of hospitalization until the date of the first occurrence of the endpoint (i.e., invasive ventilator support or all-cause in-hospital mortality); in the presence of both events, the latter date was the date of the first event. For each survival outcome, data were censored at the date of hospital discharge for patients who did not experience the events of interest during their follow-up.

We compared survival curves for mortality and composite endpoint using Kaplan–Meier estimates and tested statistical significance using the log rank test. Univariate and multivariate Cox proportional hazard models were used to evaluate the estimate of the hazard ratios (HRs) and 95% confidence intervals (CIs) for the association between all variables and the two survival endpoints. Variables that were associated with composite outcome or death (two-sided *p*-value <0.05) in the univariate analysis were included in the multivariate Cox proportional hazards models. A two-sided *p*-value <0.05 was considered to be significant. We reported two models for the analyses of the mortality and composite endpoints: model 1: All of the significant variables in the univariate analysis were included as covariates, plus gender—a known risk factor for lethality [13]; model 2: Covariates were chosen by the backward selection method. A sensitivity analysis was performed with the aim of evaluating the effect of cortisone on the two outcomes, comparing the survival curves stratified by cortisone use and evaluating the inclusion of cortisone use in the multivariable models.

The assumption of a proportional hazard was checked with the log [log(survival)] plot and by the time-dependent covariate test. We performed a repeated-measures analysis of the clinical parameters measured at baseline and during follow-up after seven days of treatment (T1), applying multivariable linear mixed models for repeated measures adjusted for treatment, time, and interaction between time and treatment with unstructured covariance structure to model within-subject errors. For each model, we reported the marginal means from each group at each time point with the *p*-value for treatment effect, time effect, or treatment-by-time interaction. For variables that were not normally distributed, geometric means were calculated from the log-transformed variable (base e). All statistical analyses were performed with SAS software, version 9.4 (SAS, Cary, NC, USA).

## 3. Results

### 3.1. Patients and Clinical Course

The data analysis was conducted by considering the treatment option adopted. Patients belonging to option A (*n* = 39) were given RAAS inhibitors (ACEIs or ARBs) and/or vasodilator agents (calcium channel blocker and alpha-1-adrenergic antagonist). All patients treated with option B (*n* = 30) received canrenone i.v. over 14 ± 11 days (mean dose 200 mg/q.d. i.v. given for at least 2 consecutive days), and eventually, ACEIs or ARBs were added. Table 1 details the treatment distribution. As reported in Table 1, the baseline features of the two groups did not differ according to general and clinical characteristics, with the exception of median PaO_2_/FiO_2_, i.e., 182 mmHg (group A) vs. 214 mmHg (group B), *p* = 0.023. Sixteen patients in group A and 17 in group B experienced hypertension, while 15 patients in group A and 15 in group B had hypokalemia.

### 3.2. Impact of Canrenone Administration

As shown in Table 2, after seven days of treatment (T1), COVID-19 patients belonging to group A (not receiving canrenone) showed a significant improvement in [K+]plasma (4.6 mmol/L, *p* < 0.001), CRP (2.2 mg/dL), IL-6 (13 pg/mL), and ΔA-aO_2_ (135 mmHg). Conversely, D-dimer was significantly increased (2224 µg/L) with no changes in mean blood pressure (BP; 93 mmHg) and ferritin levels (1109 ng/mL). Patients in group B (receiving canrenone) showed an improvement in blood pressure (meanBP, 88 mmHg), [K+]plasma (mean of 4.7 mmol/L), gas exchange (geometric mean of PaO_2_/FiO_2_ = 272 mmHg and of ΔA-aO_2_ = 107 mmHg), and lab tests (CRP = 0.6 mg/dL, IL-6 = 6 pg/mL, ferritin = 1021 ng/mL, and D-dimer = 1164 µg/L). All changes were statistically significant (*p* < 0.001) except for ferritin and D-dimer.

Overall, MRA treatment was associated with a reduced risk of both composite outcome and mortality: HR = 0.25, 95% CI 0.07–0.87 (*p* = 0.030) and HR = 0.08, 95% CI 0.01–0.48 (*p* = 0.006), respectively (Table 3).

**Figure 1:**
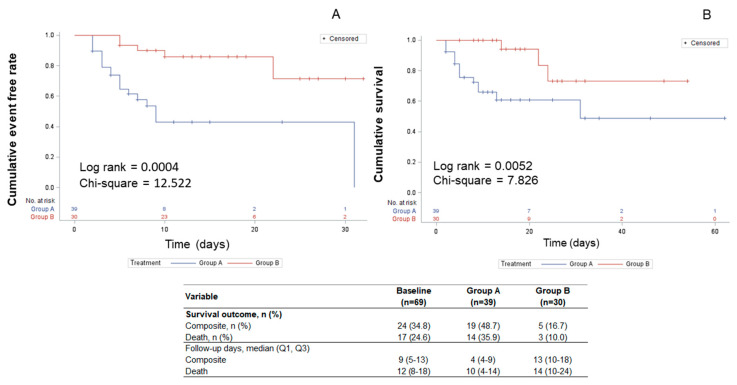
Kaplan–Meier analysis for composite outcome (**A**) and mortality (**B**). Cumulative event-free rate and survival are represented (group A, stippled line; group B continuous line). The respective log rank and chi-square values are reported.

Univariate analysis for composite outcome and death is reported in Table S1. Finally, Figure 1 shows an event-free rate of 80% (24/30) with a survival rate of 87% (26/30) across a period of 5 to 25 days post-hospitalization. In the absence of canrenone, the event-free rate was 51% (21/39) and the survival rate was 64% (25/39) across a period of 2 to 31 days post-hospitalization. Concerning composite outcomes (group A 6/30 and group B 18/39), the Kaplan–Meier analysis showed a log rank of 0.0004 (Figure 1A). Relative to mortality (group A 4/30 and group B 1/39), the log rank was 0.0052 (Figure 1B). The use of cortisone was not associated with either composite outcome or death (Figure S2). Moreover, as shown in the sensitivity analysis (Table S2), its inclusion in the multivariable model did not affect the results.

## 4. Discussion

The novelty of our study relies on the independent positive impact of the MRA treatment on all-cause mortality and clinical improvement in COVID-19 patients. Our data are in line with the recently reported evidence suggesting that aldosterone levels may reflect the clinical outcomes in COVID-19 patients: The most severe patients who required at least intensive care had significantly higher plasma levels of aldosterone than those hospitalized in medical units [12]. Moreover, as demonstrated by our group, COVID-19 patients are less capable of counteracting the progressive activation of the Ang II/AT1R axis [9]. Canrenone, the principal active metabolite of spironolactone, is considered the fourth-line therapy for hypertension in patients already treated with multiple medications [14]. When given to COVID-19 patients, they suddenly suffered a hypertensive rise. Canrenone improved outcomes, which is an effect independent of other therapies (i.e., ACEIs, ARBs, calcium channel blocker, alpha-1-adrenergic antagonist, hydroxychloroquine, lopinavir/ritonavir, anakinra, cortisone). Patients treated with canrenone did not develop hyperkalemia (>5.3 mmol/L). This favorable response may rely on the activity of canrenone, which can antagonize the disequilibrium of the RAAS pathway. The mechanisms leading to RAAS toxicity also include (i) modulation of the production of pro-inflammatory status, potentially leading to recruitment of mono/macrophages; (ii) induction of fibrosis (through AT1R); (iii) induction of vascular toxicity and modulation of angiogenesis [15]. Of note, among the RAAS-inhibiting agents, only canrenone (potassium canreonate), the active metabolite of spironolactone, can be administered intravenously [16].

Canrenone may exert some of its positive effects following the theory that spironolactone may provide protection against SARS-CoV-2-induced acute respiratory distress syndrome through different mechanisms: (i) By increasing the plasma levels of circulating ACE2, thereby limiting its attachment to cellular ACE2, (ii) by downregulating the testosterone-mediated expression of transmembrane serine protease 2 (TMPRSS2), and (iii) by a direct anti-inflammatory and antiviral effect that could avoid pulmonary complications related to COVID-19 (discussed in [17,18,19]).

Normalization of CRP and D-dimer upon canrenone i.v. is consistent with the knowledge that MRA antagonists block the inflammatory activity of aldosterone. Despite the fact that patients not receiving MRA treatment presented a reduction in CRP levels, D-dimer was found to be higher at T1. The positive effects of aldosterone receptor blockers are not confined to the kidney, as they are located in other tissues such as the endothelium, heart, hippocampus, and, in particular, lymphomonocytes [20]. A rise in aldosterone levels generally leads to the development of inflammation, fibrosis, and endothelial dysfunction. Indeed, MRA inhibits cardiac and vascular collagen turnover, reduces sympathetic activity, and improves endothelial dysfunction [8]. COVID-19 leads to disproportionate endothelial damage that disrupts pulmonary vasoregulation, promotes ventilation–perfusion mismatch, and fosters thrombogenesis [21]. Endothelial dysfunction shifts the vascular equilibrium towards vasoconstriction thus favoring organ ischemia, tissue oedema, and a pro-coagulant state [22].

Another important aspect related to canrenone treatment was the stabilization of D-dimer between admission and T1 (Figure S1). Elevated D-dimer levels constitute a significant independent biomarker of a poor prognosis in COVID-19 patients [23]. As reported in a recent meta-analysis, the rise in D-dimer was positively related to adverse outcomes (odds ratio (OR) = 4.39, 95% CI 1.85–10.41) and death (OR = 4.40, 95% CI 1.10–17.58) [24]. Increased circulating D-dimer concentrations reflect the activation of immunothrombosis by various mechanisms (e.g., local elevations in proinflammatory cytokines), vessel wall tissue damage with tissue factor production, and direct injury to small vessels [25].

Relative to the use of ARBs in COVID-19 patients with hypertension, in-patient use of ACEIs/ARBs was associated with lower risk of all-cause mortality compared with ACEIs/ARBs non-users [26], or at least was not associated with an increased likelihood of a positive SARS-CoV-2 test [5,27]. Further evidence supporting the use of RAAS in COVID-19 patients comes from Cannata et al., showing that the in-hospital continuation of ACEIs/ARBs medications was associated with a lower risk of all-cause mortality [28].

A further aspect worth mentioning is the sex-related differences in the response to SARS-CoV-2 infection. Our data showing that males have a higher risk of death (HR = 5.17, 95% CI 1.07–24.9, *p* = 0.041) are in line with the observation that among COVID-19 patients, men have a significantly higher mortality than women, a difference not completely explained by the higher prevalence of comorbidities in men [13]. Indeed, SARS-CoV-2 infection is likely to be androgen-mediated [29], possibly explaining the disproportioned mortality rate among men [30]. In this context, the hypothesis of an anti-androgen therapy to reduce the risk of developing severe symptoms following COVID-19 infection could be of interest. This concept was brought to light by Goren et al., who suggested that androgen expression might be a clue to COVID-19 severity. The authors observed that 71% of Caucasian males with a diagnosis of bilateral SARS-CoV-2 pneumonia had male androgenic alopecia, a feature dependent on genetic variants in the androgen receptor [31]. Although, canrenone and the potassium canrenoate avoid the formation of intermediate products with anti-androgenic and progestational actions [32], canrenone, in the same way as spironolactone, has been used successfully in hyperandrogenic conditions in women such as hirsutism [33,34]. However, to confirm or deny the efficacy of an androgen therapy to modulate COVID-19 severity, randomized controlled trials with bicalutamide (NCT04374279), degarelix (NCT04397718), and spironolactone (NCT04345887) are ongoing.

Finally, despite the small population number that could be acknowledged as a limitation of this study, our conclusions are based on the achieved number of events required (at least 15 cases). The overall sample size of 69 achieved a 90% power to test, with a two-sided test, whether the HR is one at a 0.05 significance level, or whether it is actually 0.20.

## 5. Conclusions

All-in-all, in COVID-19 patients with moderate-to-severe disease, MRA could be part of an optimized treatment for individuals that develop hypertension and/or hypokalemia. Based on the recent evidence that describes SARS-CoV-2 as capable of inducing endothelial damage, the present findings open new perspectives supporting the use of MRA in COVID-19 patients. Thus, target randomized clinical trials are eagerly awaited to confirm or deny our hypothesis.

## Figures and Tables

**Table 1:** Patients’ general characteristics, clinical parameters, and treatment distribution at baseline.

Characteristics	Overall Population (*n* = 69)	Group A (*n* = 39/69)	Group B (*n* = 30/69)	Group A vs. Group B	Cohen’s d
General characteristics					
Sex, m (%)	50 (72)	26 (67)	24 (80)	0.219 ^c^	
Age, years, mean ± SD	61 ± 12	60 ± 12	62 ± 12	0.311 ^w^	0.13
Hypertension, n (%)	31 (45)	18 (46)	13 (43)	0.815 ^c^	
Dyslipidemia, n (%)	14 (20)	8 (21)	6 (20)	0.958 ^c^	
Obesity, n (%)	16 (23)	9 (23)	7 (23)	0.980 ^c^	
Cardiovascular disease, n (%)	8 (12)	5 (13)	3 (10)	1.000 ^§^	
Clinical assessment at baseline					
PaO_2_/FiO_2_, mmHg, median (IQR)	190 (99)	182 (110)	214 (116)	0.023 *	0.47
ΔA-aO_2_, mmHg, median (IQR)	219 (102)	234 (151)	197 (86)	0.133 *	0.40
meanBP, mmHg, mean ± SD	97 ± 13	97 ± 13	96 ± 13	0.406 ^w^	0.03
[K+]plasma, mmol/L, mean ± SD	4.1 ± 0.5	4.2 ± 0.6	4.0 ± 0.4	0.111 ^w^	0.38
CRP, mg/dL, median (IQR)	12 (10)	11 (10)	13 (10)	0.186 *	0.28
IL-6, pg/mL, median (IQR)	83 (91)	88 (139)	83 (64)	0.757 *	0.16
D-dimer, µg/L, median (IQR)	992 (1079)	939 (890)	1057 (1135)	0.215 *	0.33
Ferritin, ng/mL, median (IQR)	1421 (2121)	1262 (2495)	1704 (1866)	0.380 *	0.02
In-hospital treatment distribution					
ACEIs or ARBs, n (%)	25 (36)	6 (15)	19 (63)	<0.001 ^c^	
Ca2+ channel blockers, n (%)	11 (16)	11 (28)	0 (0.0)	0.002 ^§^	
alpha-1-adrenergic antagonist, n (%)	4 (6)	4 (10)	0 (0.0)	0.127 ^§^	
Hydroxychloroquine, n (%)	69 (100)	39 (100)	30 (100)	-	
Lopinavir/Ritonavir, n (%)	24 (35)	23 (59)	1 (3)	<0.001 *	
Anakinra, n (%)	20 (29)	9 (23)	11 (37)	0.217 *	
Cortisone, n (%)	24 (35)	10 (26)	14 (47)	0.069 *	

SD, standard deviation; PaO_2_/FiO_2_, partial pressure of oxygen to inspiratory fraction of oxygen ratio; IQR, interquartile range; ΔA-aO_2_, alveolar–arterial oxygen gradient; meanBP, mean blood pressure; [K^+^]plasma, plasma concentration of potassium; CRP, C-reactive protein; IL-6, interleukin 6; ACEIs, inhibitors of angiotensin converting enzyme; ARBs, angiotensin receptor antagonists. ^c^ *p*-value from Pearson’s chi-square test. ^w^ *p*-value from Welch’s *t*-test. ^§^ *p*-value from Fisher’s exact test. * *p*-value from the Mann–Whitney *U*-test. Cohen’s d effect size for unequal group size.

**Table 2:** Repeated measures analysis comparing changes in variables from baseline to follow-up (T1).

Dependent Variable	Treatment	Mean Baseline	Mean T1	*p*–Value of Comparison Baseline vs. T1	Overall *p*-Value	
	Group	(95% CI)	(95% CI)			
ΔA-aO_2_	A	223 (194–255)	135 (92–198)	**0.014**	Time	**<0.001**
	B	195 (167–228)	107 (71–162)	**0.006**	Treatment	0.241
					Time * treatment	0.735
PaO_2_/FiO_2_	A	171 (152–192)	194 (155–242)	0.261	Time	**0.022**
	B	209 (183–239)	272 (213–345)	**0.034**	Treatment	**0.011**
					Time * treatment	0.420
meanBP	A	96 (92–100)	93 (89–98)	0.429	Time	**0.021**
	B	95 (91–100)	88 (83–94)	**0.018**	Treatment	0.279
					Time * treatment	0.187
[K+]plasma	A	4.2 (4–4.4)	4.6 (4.4–4.8)	**0.005**	Time	**<0.001**
	B	4.0 (3.8–4.2)	4.7 (4.5–4.9)	**<0.001**	Treatment	0.684
					Time * treatment	0.188
CRP	A	8.7 (6.7–11.2)	2.2 (1.1–4.4)	**<0.001**	Time	**<0.001**
	B	12.3 (9.2–16.5)	0.6 (0.3–1.2)	**<0.001**	Treatment	0.083
					Time * treatment	**0.002**
IL–6	A	81 (57–116)	13 (4–43)	**0.005**	Time	**<0.001**
	B	71 (48–103)	6 (3–12)	**<0.001**	Treatment	0.1852
					Time * treatment	0.3792
D–dimer	A	884 (607–1287)	2224 (1325–3731)	**0.003**	Time	0.112
	B	1426 (929–2189)	1164 (651–2083)	0.547	Treatment	0.745
					Time * treatment	**0.014**
Ferritin	A	1259 (881–1800)	1109 (664–1852)	0.668	Time	0.375
	B	1328 (884–1996)	1021 (588–1772)	0.416	Treatment	0.952
					Time * treatment	0.755

Means are estimated from multivariable linear mixed models for repeated measures adjusted for treatment, time, and interaction between time and treatment. Parameters marked with asterisks were log-transformed and the geometric mean is reported. Values in bold represent statistical changes. Time * treatment stands for interaction between time and treatment.

**Table 3:** Multivariate analysis for composite outcome and death.

Variable	HR for Death (95% CI)	*p*–Value	Variable	HR for Composite Outcome (95% CI)	*p*–Value
**Model 1**					
Treatment Group (B vs. A)	0.08 (0.01–0.48)	**0.006**	Treatment Group (B vs. A)	0.25 (0.07–0.87)	**0.030**
Age, years	1.09 (1.02–1.17)	**0.012**	Sex (male vs. female)	1.09 (0.44–2.71)	0.854
Sex (male vs. female)	5.17 (1.07–24.9)	**0.041**	Dyslipidemia (yes vs. no)	2.09 (0.88–4.98)	0.095
Hypertension (yes vs. no)	2.78 (0.73–10.6)	0.133	PaO_2_/FiO_2_, mmHg	1 (0.99–1)	0.180
Dyslipidemia (yes vs. no)	1.18 (0.32–4.4)	0.803	ACEIs or ARBs (yes vs. no)	0.58 (0.18–1.92)	0.373
Obesity (yes vs. no)	4.83 (1.35–17.3)	**0.016**	Lopinavir/Ritonavir (yes vs. no)	1.21 (0.45–3.24)	0.712
PaO_2_/FiO_2_, mmHg	1 (0.99–1.01)	0.299			
Lopinavir/Ritonavir (yes vs. no)	0.83 (0.18–3.86)	0.815			
**Model 2**					
Treatment Group (B vs. A)	0.07 (0.04–0.53)	**<0.001**	Treatment Group (B vs. A)	0.19 (0.07–0.53)	**0.002**
Age, years	1.1 (1.04–1.16)	**0.002**	Dyslipidemia (yes vs. no)	2.52 (1.08–5.89)	**0.032**
Sex, (male vs. female)	7.8 (1.88–32.6)	**0.005**			
Hypertension (yes vs. no)	3.72 (1.19–11.61)	**0.024**			
Obesity (yes vs. no)	3.39 (1.22–9.41)	**0.019**			

Model 1 included all of the significant variables in the univariate analysis, plus gender. Model 2 included all of the variables selected using the backward selection method. HR: hazard ratio. Values in bold represent statistical changes.

## Supplementary Materials

The following are available online at https://www.mdpi.com/2077-0383/9/9/2943/s1, Figure S1: Changes of D-dimer averages from baseline to T1, in treatment groups A and B. Figure S2: Kaplan–Meier analysis for composite outcome. Table S1: Univariate analysis for composite outcomes and death. Table S2. Sensitivity multivariate analysis for composite outcome and death, evaluating the inclusion of cortisone as a covariate.
