# Supplementary material for: The Efficacy of the Mineralcorticoid Receptor Antagonist Canrenone in COVID-19 Patients
Source: J Clin Med. 2020 Sep 11;9(9):2943. doi: 10.3390/jcm9092943 (PMC7564548; doi:10.3390/jcm9092943)
Supplement: Supplementary file 1 [file jcm-09-02943-s001.pdf]

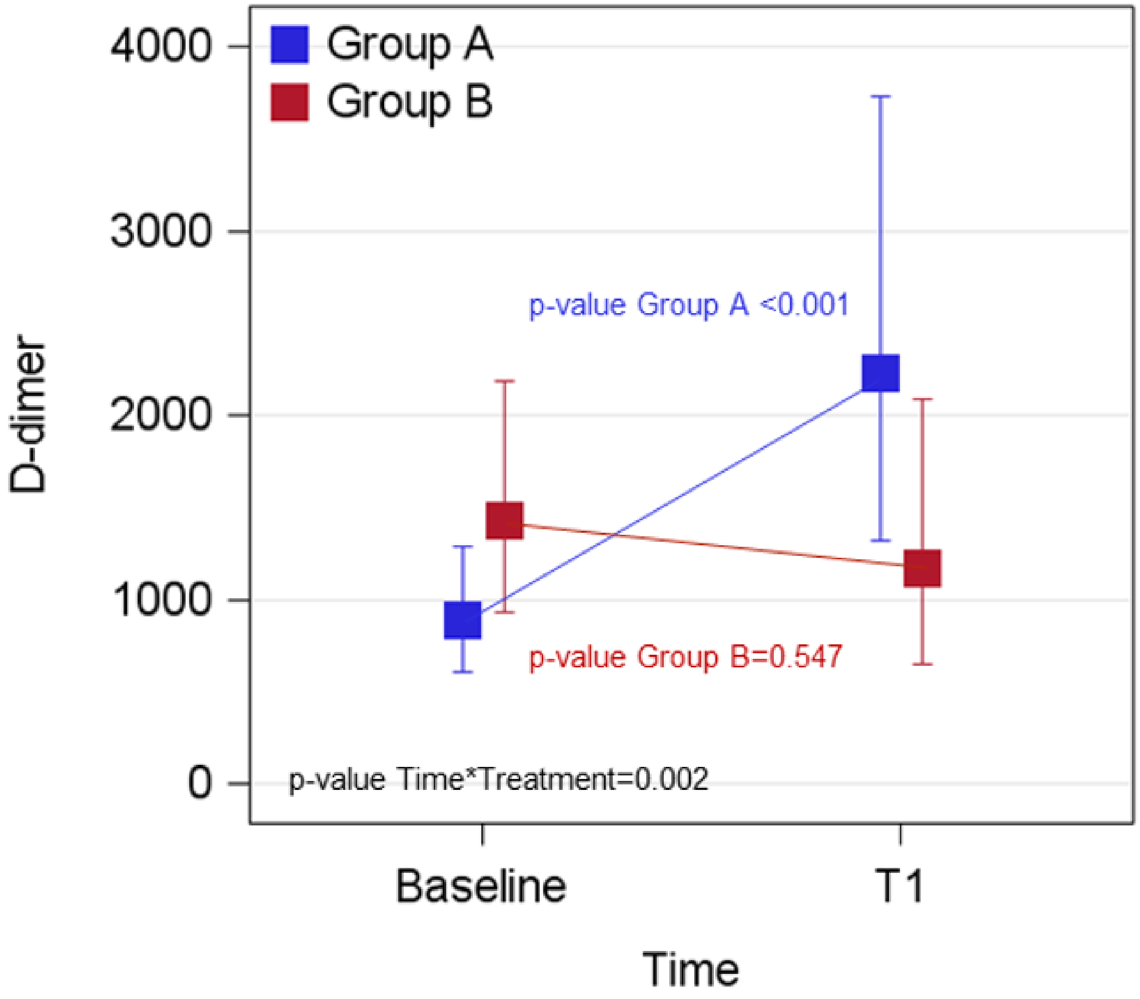

Figure S1. Changes of D-dimer averages from baseline to T1, in treatment groups A and B. Means are estimated from multivariable linear mixed models for repeated measures adjusted for treatment, time, and interaction between time and treatment. Geometric mean of CRP and D-dimer were reported.

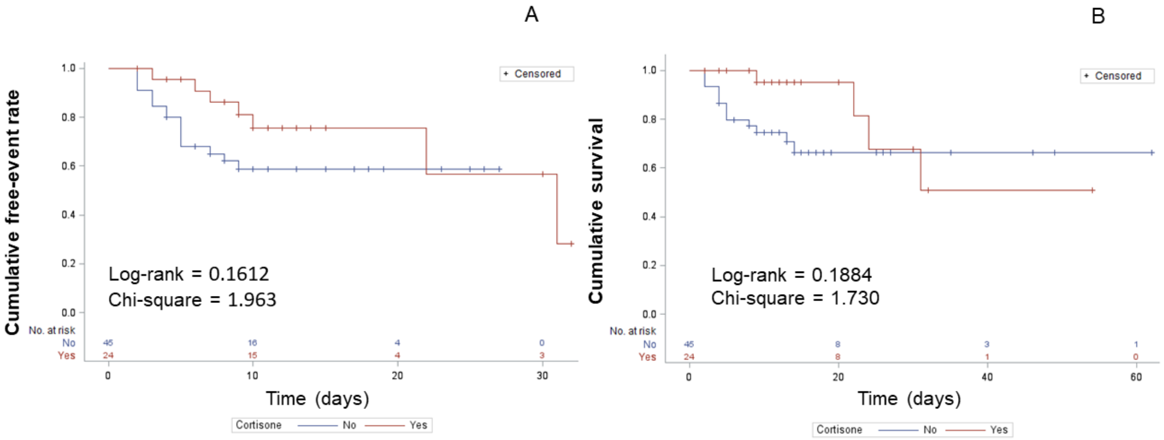

Figure S2. Kaplan-Meier analysis for composite outcome (panel A) and mortality (panel B), stratified by cortisone use. Cumulative free-event rate and survival are represented. The respective Log Rank and Chi-square are reported.

Table S1. Univariate analysis for composite outcome and death.

Variable	HR for death (95% CI)	p-value	HR for composite outcome (95% CI)	p-value
Treatment Group, B vs A	0.2 (0.06 - 0.71)	**0.012**	0.2 (0.07 - 0.54)	**0.002**
Sex, M vs F	1.7 (0.53 - 5.43)	0.374	0.85 (0.35 - 2.06)	0.715
Age, years	1.08 (1.02 - 1.13)	**0.004**	1.04 (1 - 1.07)	0.052
Hypertension, yes vs no	3.47 (1.22 - 9.88)	**0.02**	1.82 (0.81 - 4.1)	0.149
Dyslipidemia, yes vs no	4.91 (1.89 - 12.78)	**0.001**	2.45 (1.06 - 5.67)	**0.036**
Obesity, yes vs no	3.59 (1.38 - 9.36)	**0.009**	1.85 (0.78 - 4.36)	0.161
Cardiovascular disease, yes vs no	2.93 (0.81 - 10.57)	0.101	1.71 (0.51 - 5.8)	0.387
PaO_2/FiO_2, mmHg	0.99 (0.98 - 1)	**0.028**	0.99 (0.99 - 1)	0.058
$\Delta A\text{-}aO_2$, mmHg	1 (1 - 1.01)	0.072	1 (1 - 1.01)	0.085
meanBP, mmHg	1.02 (0.98 - 1.06)	0.31	1.01 (0.98 - 1.04)	0.575
[K+]plasma, mmol/L	1.11 (0.41 - 3.02)	0.836	0.86 (0.36 - 2.07)	0.736
CRP, mg/dL	1 (0.94 - 1.06)	0.924	1.03 (0.98 - 1.08)	0.317
IL-6, pg/mL	1 (1 - 1)	0.967	1 (1 - 1.01)	0.221
D-dimer, mg/L	1 (1 - 1)	0.489	1 (1 - 1)	0.363
Ferritin, ng/mL	1 (1 - 1)	0.491	1 (1 - 1)	0.676
ACEi or ARBs, yes vs no	0.43 (0.14 - 1.33)	0.144	0.25 (0.09 - 0.75)	**0.013**
Ca2+ channel blockers, yes vs no	0.5 (0.11 - 2.25)	0.365	2.35 (0.9 - 6.1)	0.08
Lopinavir/Ritonavir, yes vs no	2.72 (1.04 - 7.11)	**0.041**	3.33 (1.42 - 7.81)	**0.006**
Anakinra, yes vs no	0.87 (0.31 - 2.47)	0.79	1 (0.43 - 2.35)	0.993
Cortisone, yes vs no	0.48 (0.16 - 1.48)	0.202	0.52 (0.21 - 1.34)	0.177

Table S2. Sensitivity multivariate analysis for composite outcome and death, evaluating the inclusion of cortisone as a covariate.

Variable	HR for death (95% CI)	p-value	Variable	HR for composite outcome (95% CI)	P-value
Treatment Group (B vs A)	0.06 (0.01 - 0.48)	**0.008**	Treatment Group (B vs A)	0.24 (0.07 - 0.82)	**0.024**
Age, years	1.09 (1.02 - 1.17)	**0.012**	Sex (male vs female)	1 (0.39 - 2.57)	0.996
Sex (male vs female)	6.03 (1.15 - 31.55)	**0.033**	Dyslipidemia (yes vs no)	2.08 (0.88 - 4.92)	0.096
Hypertension (yes vs no)	2.57 (0.66 - 9.92)	0.172	PaO_2/FiO_2, mmHg	1 (0.99 - 1)	0.157
Dyslipidemia (yes vs no)	1.12 (0.29 - 4.31)	0.872	ACEi_ARBs (yes vs no)	0.59 (0.18 - 1.95)	0.385
Obesity (yes vs no)	5.13 (1.38 - 19.01)	**0.015**	Lopinavir/Ritonavir (yes vs no)	1.06 (0.37 - 3)	0.917
PaO_2/FiO_2, mmHg	1 (0.99 - 1)	0.316	Cortisone (yes vs no)	0.71 (0.25 - 2.02)	0.517
Lopinavir/Ritonavir (yes vs no)	0.9 (0.19 - 4.23)	0.897			
Cortisone (yes vs no)	1.5 (0.32 - 6.97)	0.604			
